# *Bacillus* *subtilis* isolates from camel milk as probiotic candidates

**DOI:** 10.1038/s41598-023-30507-0

**Published:** 2023-02-28

**Authors:** Roya Daneshazari, Mohammad Rabbani Khorasgani, Afrouzossadat Hosseini-Abari, June-Hyung Kim

**Affiliations:** 1 Department of Cell and Molecular Biology and Microbiology, Faculty of Biological Science and Technology, University of Isfahan, Isfahan, Iran; 2 Department of Chemical Engineering, College of Engineering, Dong-a University, Busan, Korea

**Keywords:** Microbiology, Applied microbiology

## Abstract

Recently *Bacillus* spp. has gained much attention as potential probiotics due to the production of resistant cells. So, this research is purposeful for evaluation of probiotic characteristics of *Bacillus* isolates from camel milk as a suitable source for growth and isolation of microorganisms that can be candidate to be used as probiotic. First, forty-eight colonies were screened by using morphological and biochemical analysis. Among the isolates, two of them were recognized as *Bacillus* *subtilis* CM1 and CM2 by partial 16SrRNA sequencing that, probiotic potentials of them were evaluated. Both of them, in the preliminary safety screening, were found negative for hemolysis and lecithinase activity. Also, in vitro characteristics such as acid, bile salts and artificial gastric juice resistant, cell surface hydrophobicity, auto-aggregation, antioxidant characteristics, and adherent capability to HT-29 cells were determined for them approximately in the range of other probiotic strains. Two strains were susceptible to various antibiotics and enterotoxigenic activities were not detected by PCR which means isolated *Bacillus* strains could be classified as safe. Altogether, results demonstrate that *Bacillus* CM1 and CM2 strains could have the potential of consideration as probiotics, however more extensive in vitro/vivo studies are needed.

## Introduction

Live microorganisms that administration of sufficient quantities of them could have beneficial healthy effects are defined as probiotics^1^. Today, owing to recognition of health benefits of probiotics as food supplements that include inhibition of intestinal pathogens by promoting the growth of healthy microflora in gastrointestinal tract, reduction in cholesterol level, control of diarrhea, immune response enhancement, anti-mutagenic and anti-carcinogenic activity, alleviation of lactose intolerance and etc., the market for probiotics has grown too^2,3^. Although two main genera *Lactobacillus* and *Bifidobacteria* are largely represented on the market as convential probiotics that mostly isolated from sources such as parts of GIT, feces, milk and fermented food products, but different species from *Streptococcus*, *Propionibacterium*, *Bacillus*, *Enterococcus* and *Saccharomyces* from various sources are claimed as probiotics too^3–6^. Also, however plenty of accessible probiotic strains are belonged to the lactic acid bacteria (LAB) as a group of non-sporulating bacteria but it is important to know that, in comparable of vegetative cells, spore forming bacteria such as *Bacillus* species due to their interesting properties have gained much attention^1,7^. A probiotic strain must fulfill some essential standards and must tolerate manufacturing, storage, transportation, application steps and so on. The extremely resistance properties of spores to heat, UV irradiation, pH conditions, desiccation and solvents offers capability of long time periods of storage at low or room temperature, higher stability in heat processing and better acid tolerance which they are important traits for overcoming to some difficulties in term of LAB usage as probiotics^4,8,9^. So, the possibility of incorporation of them in food products can be raised and could be dominant microorganisms in pasteurized milk-based products^5,10^. Although several *Bacillus* strains with probiotic potential have been evaluated in various in vitro and in vivo studies^9^ but some of them such as *B.* *subtilis,* *B.* *polyfermenticus*, *B.* *clausii*, *B.* *coagulans*, *B.* *licheniformis* and *B.* *pumillus* have been approved for commercial use as dietary supplements or growth promoters in aquaculture and in animals respectively, and much effort has been devoted to research on the *Bacillus* isolation from various sources for probiotic products development^5,11,12^. Bacteria of *Bacillus* genus specially *B.* *subtilis* are dominant in soil, but they have been identified in water, air, human and animal gut, vegetables, fermented foods, raw and pasteurized milk and dairy products^4^. Thus, owing to their ubiquitous in different environment, they could easily find their way in food products and are often present in milk microflora^5^.

In many countries of dry land and desert ecosystems, camels due to high adaptation to the hostile climatic conditions, have significant role in life of these types of communities by providing meat, milk and transportation^13^. In addition, camels have medical importance through their milk and urine^14^. Camel milk has good nutritional and medicinal properties and as a medicinal drink in Middle Eastern, Asian and African cultures has been used. Camel milk is reputed as an anti-diabetic, anti-cancerous and anti-infectious food and the therapeutic effects of camel milk have been investigated in case reports, in vitro or in vivo studies and clinical trials^15^. Camel milk, in some aspects is different from other ruminant milk. It contains all the essential nutritious needed for humans and its biochemical composition is close to human milk thus it can be served as alternative of cow milk^16^. It has many groups of water and fat-soluble vitamin which the vitamins and iron content of it, is 3times and 10folds higher than in cow milk respectively^15,17^ and because of high vitamin C content, camel milk has powerful antioxidant activity^18^. It contains a large amounts of various proteins like albumin, immunoglobulins and lactoferrin^19,20^ that are apparently more heat resistant than those of cowʼs milk^17^. It is also rich in amount and type of amino acids such as valine, methionine, lysine, arginine and phenylalanine^19^. The milk has low sugar, low fat content, high minerals especially zinc, calcium and kalium and large concentration of insulin^15,21^. Another advantage of this milk, is its low allergenicity^18^. Aside from the high nutritional value and physicochemical characteristics of milk, a rich bacterial diversity exists in the milk and fermented products that have been received low attention, also few researches have been reported about isolation of strains with probiotic potential from them^22^. so, this research was aimed for assessment of probiotic properties of *B.* *subtilis* strains from camel milk.

## Results:

### Strain isolation and molecular identification

**Figure 1:**
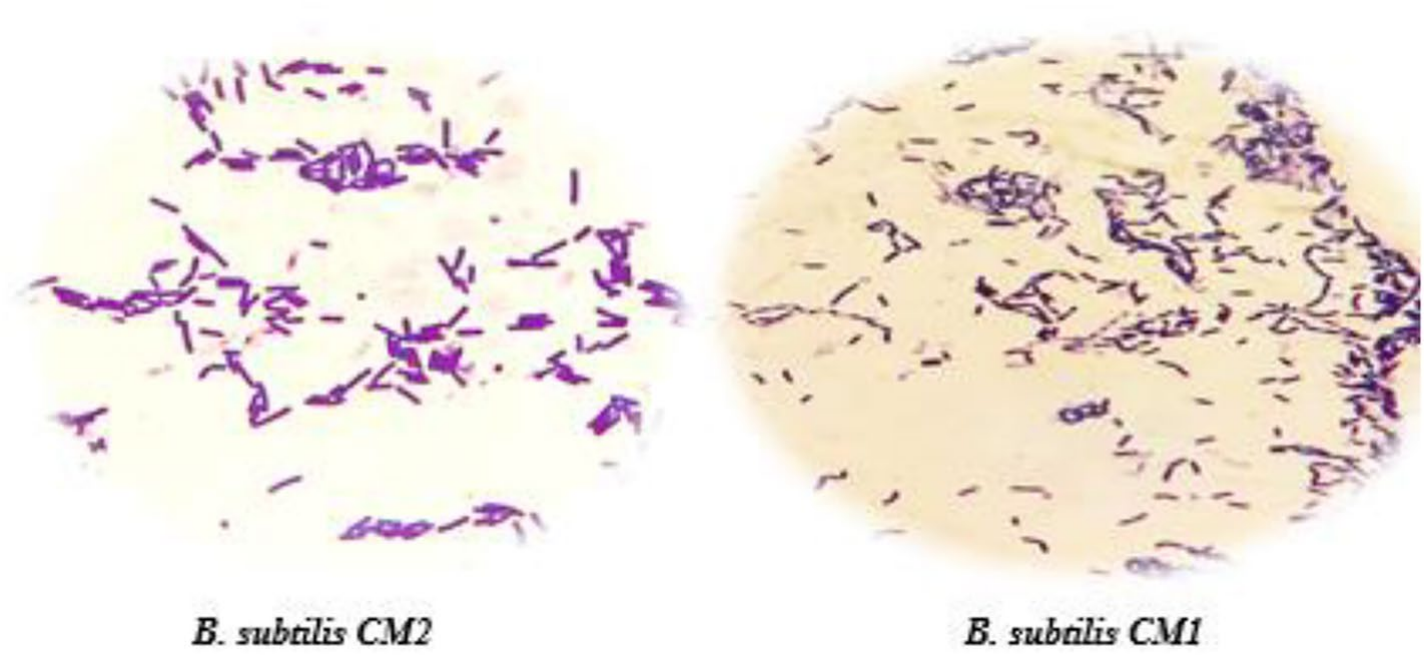
Gram staining reaction of bacteria isolates.

**Figure 2:**
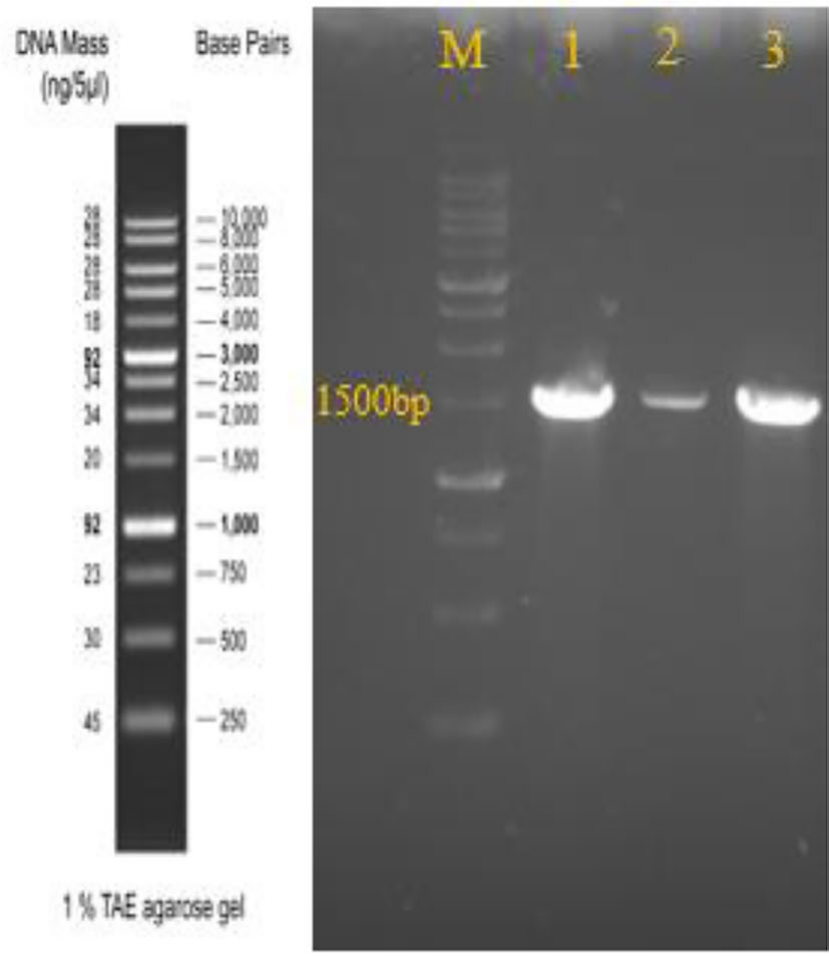
PCR products of 16SrRNA from *Bacillus* isolates. M: 1 Kb DNA ladder. Lane 1: Positive control. Lane 2 and 3: CM1 and CM2 strains.

Initially, 48 bacterial colonies were isolated from milk samples and primarily identification of them was performed by morphological and biochemical tests (Fig. 1, Table 1). Selected isolates were Gram positive, catalase, citrate, nitrate reduction and motility positive and had a shape of bacilli containing oval spores in the center or subterminal and were able to hydrolyze mannose, glucose, maltose and starch were known as *Bacillus* genus. Then among spore- forming isolates, two isolates designated as CM1 and CM2 were selected for molecular identification. 16SrRNA gene sequence analysis of these isolates revealed that these bacteria belonged to the *B.* *subtilis* and sequences of the 16SrRNA gene from the two isolates of *B.* *subtilis* were deposited in the NCBI database (national center of Biotechnology Information) with the accession numbers MK559537 and MK611084 (Fig. 2, Table 2). https://www.ncbi.nlm.nih.gov/nuccore/MK611084.1https://www.ncbi.nlm.nih.gov/nuccore/MK559537.1.

**Table 1:** Biochemical characterization of *Bacillus* isolates from camel milk.

Tests	CM1	CM2	Tests	CM1	CM2
Gram staining	+	+	Glucose	+	+
Spore formation	+	+	Maltose	+	+
Starch hydrolysis	+	+	Mannose	+	+
Simon ʼs citrate	+	+	Nitrate reduction	+	+
Methyl red	−	−	VP	+	+
Catalase	+	+	Growth at 50 °C	+	+
Urease	−	−	SIM	−/−/+	−/−/+

Plus (+) and minus sign (−) indicate the positive and negative results of reaction/test, respectively.

**Table 2:** Accession numbers and similarity of *Bacillus* species isolated from camel milk.

Bacteria	Accession number	Query coverage	percent identity
*Bacillus* *subtilis* strain CM1	MK559537	99%	99.65%
*Bacillus* *subtilis* strain CM2	MK611084.1	100%	99.30%

### Hemolytic and Lecithinase activity of isolates

Both of the selected bacterial isolates showed α-hemolysis pattern. Also, these two bacteria were lecithinase negative while B*.* *cereus* was lecithinase positive (Table 3).

**Table 3:** The culture results for selected isolates from camel milk in different conditions.

Bacteria	Growth at					
	pH4	Gastric juice tolerance	pH2	Bile 0.3%	Lecithinase activity	Hemolytic activity
*B.* *subtilis* Strain CM1	87.62 ± 0.86^a^	73.58 ± 0.68^a^	88.58 ± 2.14^a^	0.35 ± 0.02^a^	–	α
*B.* *subtilis* strain CM2	95.22 ± 1.21^b^	74.47 ± 0.63^a^	86.64 ± 0.78^a^	0.37 ± 0.03^a^	–	α

Data for three replications are presented as mean ± SD. Mean within the same column followed by different supercript letters differ significantly (P < 0.05).

### Tolerance of isolates to acid and bile

Tolerance of two obtained *B.* *subtilis* strains to low pH (pH 2 and pH 4) and 0.3% bile salts was evaluated. Approximately more than 80% of bacterial populations of two strains survived at acidic conditions, so tolerance of these isolates was high. Also, both of them showed resistance to bile salts (Table 3).

### Gastric juice tolerance assay

The isolates were investigated for artificial gastric juice tolerance by determination of total viable cell count at 0 and 4 h after exposure to gastric conditions. The viability count of more than 70% indicated that these two strains could be survived after 4 h and have the ability to pass through stomach conditions (Table 3).

### Determination of cell surface properties

Surface characteristics were evaluated based on auto-aggregation, adhesion capacity to HT-29 cells and hydrophobic traits. In order to determine colonization quality, the bacterial adhesion ability to hydrocarbons (Chloroform, Ethyl acetate and Toluene) was assessed that results are reported in Table 4. Regarding to auto-aggregation, this attribute for two strains varied from 39% (CM2) to 49% (CM1) but in comparison, about 62% cells aggregation were showed for isolates after 24 h. Also, the isolates showed adhesion characteristics to colonic adenocarcinoma cells and *B.* *subtilis* CM1 and *B.* *subtilis* CM2 strains were adhered 49.66% and 47.35% to the HT-29 cells respectively (Table4).

**Table 4:** Percentage of auto-aggregation, cell attachment and hydrophobicity traits of isolates.

Strain	Auto-aggregation (%)		Hydrophobicity(%)			
	4 h	24 h	Chloroform	Toluene	Ethyl acetate	HT-29 attachment
*B.* *subtilis* strain CM1	48.78 ± 5.55^a^	62.07 ± 2.07^a^	55.00 ± 1.80^a^	56.24 ± 2.58^a^	42.44 ± 2.94^a^	49.66 ± 0.82^a^
*B.* *subtilis* strain CM2	39.10 ± 1.74^b^	62.48 ± 0.31^a^	48.96 ± 1.18^b^	60.93 ± 1.33^b^	60.73 ± 0.67^b^	47.35 ± 0.02^b^

Cell surface characteristics are presented as mean ± SD of three replications. Isolates having different superscript letters differ significantly (P < 0.05).

### Antibiotic susceptibility

The inhibition zone of selected antibiotics was revealed the antibiotic sensitivity of *B.* *subtilis* CM1 and CM2 to several antibiotics which are presented in Table 5.

**Table 5:** Antibiotic susceptibility and antioxidant activity of the *Bacillus* isolates.

Strain	Chloramphenicol	Tetracycline	Erythromycin	Streptomycin	Vancomycin	Gentamycin	Clindamycin	Penicillin
*B.* *subtilis* CM1	34.00 ± 3.61^**S**^	30.33 ± 1.53^**S**^	27.33 ± 2.08^**S**^	17.00 ± 1.00^**S**^	21.33 ± 1.53^**S**^	27.33 ± 1.53^**S**^	18.33 ± 0.57^**S**^	30.66 ± 3.05^**S**^
*B.* *subtilis* CM2	37.00 ± 2.08^**S**^	34.66 ± 3.05^**S**^	24.33 ± 2.51^**S**^	20.33 ± 1.53^**S**^	23.67 ± 1.53^**S**^	30.33 ± 1.53^**S**^	22.67 ± 1.15^**S**^	32.67 ± 2.08^**S**^

Mean ± SD expressing data of inhibition diameter (mm) in three replications. Sensitive (s), Resistance (r).

### Antioxidant activity

DPPH scavenging activity was done for determination of the antioxidant nature of *Bacillus* strains and antioxidant activity recorded 33.8 ± 1.37% and 42.39 ± 2.59% for cell- free supernatant of CM2 and CM1 respectively.

### Detection of enterotoxin genes

**Figure 3:**
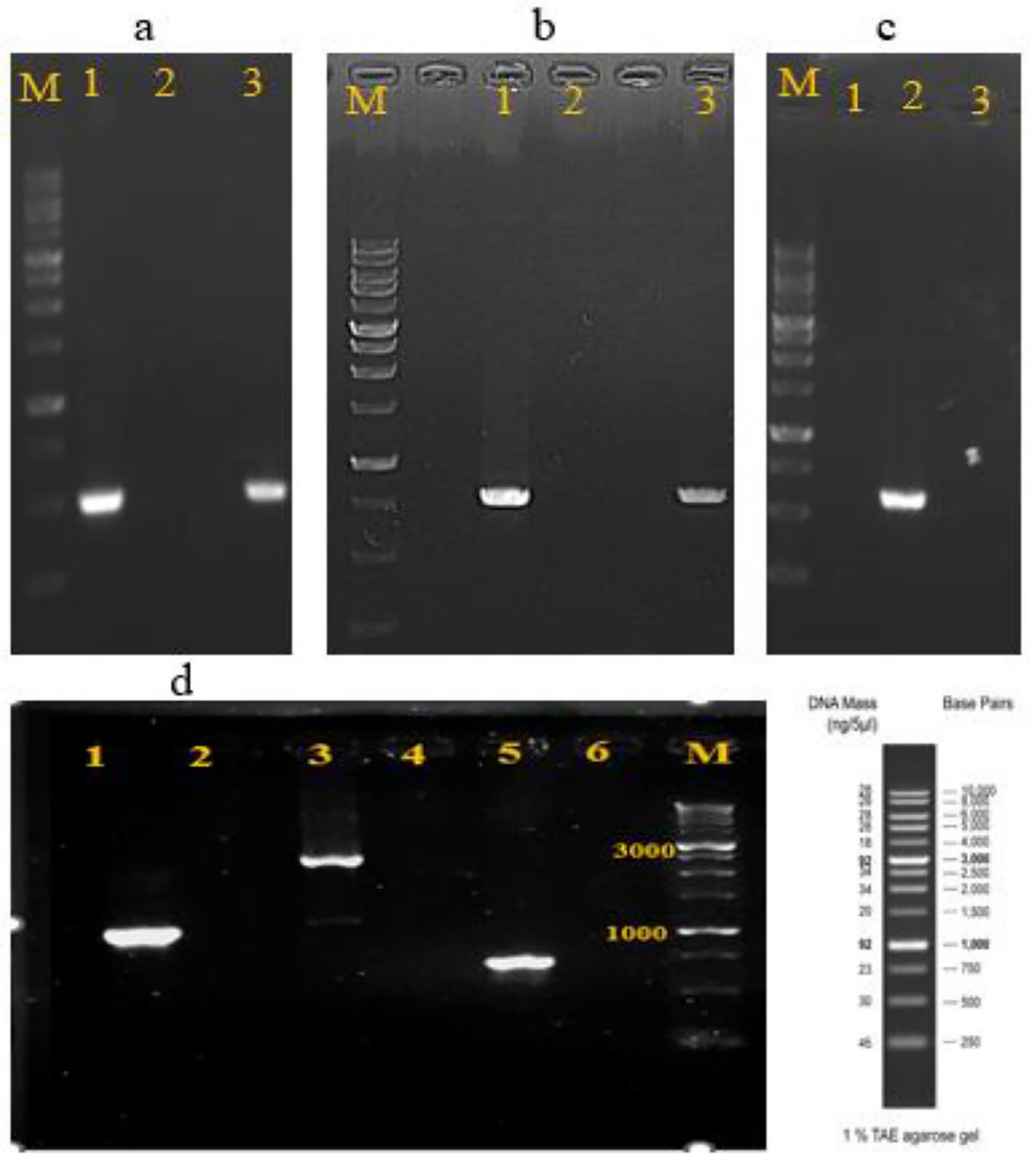
PCR products of enterotoxin genes from the isolated *Bacillus* *subtilis* strains and *Bacillus* *cereus*. Lane M: 1 kb DNA ladder. (**a**) nhe A gene 1: positive control, 2: CM1, 3: CM2. (**b**) nheB gene 1: positive control, 2: CM1, 3: CM2. (**c**) nheC gene 1: CM1, 2: positive control, 3: CM2. (**d**) 1, 3 and 5: hblA, hblB and hblC genes from positive control, 2,4 and 6: negative results of hbl genes for CM1 (negative results observed for both of CM1 and CM2 strains).

This test in order to evaluation of safety of strains was done based on PCR. *B.* *subtilis* CM1 did not carry non-hemolytic enterotoxin (nhe) and hemolysin (hbl) genes but in contrast, *B.* *subtilis* CM2 was found to carry nheA and nheB genes. Also, we could detect all six enterotoxin genes in positive control (Fig. 3).

## Discussion

Camel milk and its products have been given much attention in the world owing to their beneficial effects such therapeutic and nutritional values^13,15^. Recent scientific reports have indicated the camel milk as a rich source for probiotics^19^ that aside from nutritional composition as well as the therapeutic and physicochemical properties of milk, information about its microbiota is limited^23^. Results of microflora diversity in camel milk satisfy to high diversity across countries^24^ that lactic acid bacteria be as a one of the predominant bacteria which isolated from it^25^. Therefore, according to the latest studies and available reports, there are no data on the isolation of *Bacillus* probiotics from camel milk So, here camel milk samples were prepared for isolation *Bacillus* strains with probiotic potentials as a new source. The isolated strains based on results of biochemical and molecular tests were related to *B.* *subtilis* and further used for probiotic evaluation.

It is noticeable to know that probiotic characteristics be strain specific that its own ability is mainly dependent on strain isolation sources and its target, thus for consideration of one microorganism as a probiotic, in vitro/vivo probiotic properties must be evaluated^26–28^. Hemolysis and Lecithinase activity are usually considered for destroying host cells and tissues^29^, so screening of bacteria for these products is important for ensuring safety of one isolate^27^. Some *Bacillus* species produced hemolysis which this capability is considered a disadvantage for probiotic strain and could be a health risk for the host^8^. In this study, both of the strains showed no lecithinase activity. Similar observations have been reported for probiotic candidates like *B.* *clausii* UBBC07 and *Bacillus* *strains* BS3 and BS31^30,31^. Among screened isolates, *B.* *subtilis* CM1 and CM2 strains display α-hemolytic activity results. Similar results were shown by Keubutornye et al.^32^. Also, Naeem et al. had worked on probiotic potential of *Bacillus* strains and their results showed no hemolysis for isolated strains^8^. Although γ-hemolytic and α-hemolytic strains are remarkable as safe that means the *Bacillus* species did not show any risk to host, but γ-hemolytic isolates are ideal for consideration and usage as probiotics^29,32^.

Assessment of the antibiotic susceptibility of bacterial cells was conducted to ensure inability of strains for transferring of antibiotic resistance determinants that is other essential aspects for investigation of probiotic safety^33^. Antibiotic resistance pattern indicated the susceptibility of *B.* *subtilis* CM1 and CM2 strains to antibiotics, that ensures their inability to possess antibiotic resistance^34^. These results were similar to previous studies about *B.* *subtilis* NC11, *B.* *subtilis* TPS4, *Bacillus* *velezensis* TPS3N and *Bacillus* *amyloliquefaciens* TPS17 which were found to be sensitive to antibiotics^32,35^.

Since, acid and bile salts in the stomach and intestine respectively, are the first biological barriers that a probiotic strain must be overcome after ingestion to reach its place of action^28^, acid and gastric juice tolerance as well as bile resistance are as a most essential factors for viability and growth of probiotic strains during their transit to the gastrointestinal tract^36^. Two isolates could tolerate the acidic pH and artificial gastric juice condition and both strains showed bile salt resistance. So, present findings show similarity to previous results about *Bacillus* strains with probiotic potential^37–40^.

Other factors that should be considered for probiotic potential are cell surface hydrophobicity, auto-aggregation and epithelial cell adherence that be required for adhesion to the target sites of gastrointestinal tract^41^. The capability of bacteria to bind to themselves in addition to binding to the extracellular matrix of host tissues or host cells known as auto-aggregation^33^. Auto-aggregation of probiotics prevents their elimination from the body and could give them a superiority trait and capability for interaction with other bacteria^27^. Moreover, to determination of cell surface properties, microbial adhesion to hydrocarbons was performed as another important property of bacteria which aids attachment of microorganisms to the intestinal epithelium. Bacterial adhesion characteristic considered as a complex process which needed bacterial cell membranes contact with interacting surfaces, so this ability offers a competitive advantage for probiotic bacteria^12,41,42^. In addition to mechanisms of interaction between the strain and the superficial components of intestinal cells, the types of cell lines (HT29 or Caco2) can also be affected the adhesion capacities of bacteria to epithelial cells^43^. In present study, two isolates exhibited auto-aggregation that increasing over time. Similar observations were obtained by Ragul et al. for *Bacillus* species and Dial et al. for *Lactobacillus* *plantarum*^40,44^. Also, the results of MATH in this research are comparatively similar to results of Lee et al. and Thirabunyamon et al. for *Bacillus* isolates^35,45^. In contrast, in a study by Kuebutornye et al., *B.* *subtilis* TPS4, *B.* *velezensis* TPS3N and *B.* *amyloliquefaciens* TPS17 were reported as *Bacillus* isolates from the gut of Nile tilapia that exhibited approximately 85 to 97% hydrophobicity measured with chloroform and 74 to 90% hydrophobicity with ethyl acetate. The higher hydrophobicity results of them in comparable of *Bacillus* isolates in our report, indicating higher electron donation (chloroform) and acceptability (ethyl acetate) of isolates, therefore better adhesion to epithelial cells comparable with *Bacillus* strains in our study^32^. The HT-29 cells attachment percentage of *B.* *subtilis* CM1 and CM2 strains were higher than *Bacillus* isolates reported by Talebi et al. on Caco_2_ cell line^37^. The results are in line with Mahmoudi et al. that introduced several levels of attachment for *Lactobacillus* isolates from camel milk on Caco2 and HT-29 MXT as various groups of cell lines^46^.

The antioxidant potential of probiotic microorganisms is another beneficial and therapeutic value of probiotics^47^. Generation of free radicals in the body cause damage to macromolecules like lipids, proteins and DNA, therefore, probiotics could neutralize free radicals with their antioxidant potential that would be beneficial for the host^48^. In this study, two selected isolates exhibited antioxidant activity relatively similar to those reported by Talebi et al. for *Bacillus* *atrophaeus* and *Bacillus* *safensis*^37^.

Also, two major complexes, the hemolysin BL (Hbl) and the non-hemolytic enterotoxin (Nhe), that cause diarrhea are noticeable as a reason for food poisoning which included *B.* *cereus*^49^. So, one of the important criteria to ensuring from safety of *Bacillus* isolates is investigation of enterotoxins production. Hbl and Nhe consist of the protein parts B, L1 and L2 codification by hbl A, hbl C and hbl D as well as Nhe A, Nhe B and Nhe C encoded by nhe A, nhe B and nhe C respectively^50^. When PCR was carried out, only *B.* *subtilis* CM2 was positive for nhe A and nhe B genes. It should be noted that combination of all three parts of the Hbl and Nhe enterotoxin complexes is required to show the enterotoxigenic traits^29^. Therefore, current results could be considered negative for two isolates and our findings could be similar to most of the studied *Bacillus* strains, they could not express all genes of enterotoxin complex together and were safe^29,49^.

Finally, it can be concluded that *B.* *Subtilis* CM1 and *B.* *subtilis* CM2 could be notable as probiotic candidates. This selection regarded based on analyzing the results of all tests that showed the strains had desirable probiotic potential, however other in vitro and in vivo evaluations including enzymatic activity, co-aggregation, antimicrobial activity, biofilm formation, cholesterol reduction and animal models must be performed in the future for the final decision about their application as probiotic strains.

## Methods

### Camelʼs milk samples preparation

The raw milk samples were gathered from Varamin (Tehran province, Iran) under aseptic condition and in accordance with the ethical principles and standards guidelines for conducting Veterinary Research in Iran, stored at 4 °C and serial dilutions were prepared in saline buffer, heated at 80 °C, 15 min. Afterward 0.1 ml of any sample was streaked on nutrient agar plates (Merck, Germany), incubated for 24 h, 37 °C and different morphological colonies were purified, checked for Gram staining and catalase activity. Finally, biochemical tests and sequencing of 16SrRNA genes was done for further identification of obtained isolates^17,37^. The animal experiments have acquired approval of Research Ethics Committees of University of Isfahan (Approval ID: IR.UI.REC.1400.024) and performed under the ARRIVE guidelines.

### Identification of *B. subtilis* isolates

First biochemical tests were used for identification of isolates as described elsewhere according to Bergey manual of Systematic Bacteriology. Then for identifying the bacteria with PCR, after 18 h incubation of the selected isolates, boiling method was used for DNA extraction from them. Respectively, 27F (5′-AGAGTTTGATCCTGGCTCAC-3′) and 1492R (5′CGGTTACCTTGTTACGACTT-3′) were used as forward and reverse primers with an expected product size of 1500 bp. Finally, the PCR products were sequenced and analyzing of the sequence was done by BLAST algorithm (NCBI)^51,52^.

### Screening of *B. subtilis* isolates for probiotic properties

#### Hemolysis activity

The selected isolates were streaked on blood agar plates (Merck, Germany) and incubated for 24 h, 37 °C. Then hemolysis pattern was classified as α, β or γ-hemolysis. *S.* *aureus* ATCC25923 was used as the control^53^.

#### Lecithinase activity

A loopfull of each strain and *B.* *cereus* ATCC14579 as positive control were streaked as a straight line on the egg yolk agar (Biomark, India) and incubated (24–48 h, 37 °C) for detection of lecithinase production^30^.

### Acid tolerance

For acid tolerance determination, several pH grades were prepared by hydrochloric acid solution (Merck, Germany) 5N to pH 2.0 and 4.0 in Phosphate-Buffered Saline (PBS). The isolates were incubated in nutrient broth (Biolife, Italy) for 18 h at 37 °C, then cell pellet was harvested and washed in PBS, resuspended in both pH solutions, including 2 and 4, and incubation was done for 4 h, 37 °C. Plate counts on nutrient agar at 0 h and 4 h were used for assessment of survivability according to this equation:

$${\text{Survival}}\,{\text{rate }}\left( \% \right) = \left( {N1/N0} \right) \times 100,$$

where N1 and N0 represent (log cfu/ml) count of selected species after and before treatment respectively^37^.

### Gastric juice tolerance

For gastric juice test, pepsin (Sigma-Aldrich, USA) (0.3 w/v) and NaCl (Merck, Germany) (0.5% w/v) was added to nutrient broth and adjusted to pH 2.5. First, strains were incubated (nutrient broth, 18 h, 37 °C), and pellet washed twice in PBS, then cell suspension was diluted in gastric juice pH 2.5 and incubated. Viable cells count was investigated at 0 and 4 h for samples.

$${\text{Viability }}\left( \% \right) \, = \left( {\log Nt/\log N0} \right) \times 100,$$

where N_0_ and Nt are initial and final viable cells (cfu/ml)^53^.

### Bile salts resistance

For assessment of bile salts resistance, 100 μl bacterial suspensions were inoculated into nutrient broth containing bile salts (Sigma-Aldrich, USA) at concentration of 0.3% and nutrient broth without salts, followed by incubation (37 °C, 8 h). Inhibition rate was calculated by recording the absorbance at 600 nm.

$${\text{C inh }} = \frac{(T8 - T0)control - (T8 - T0)treatment}{{(T8 - T0)control}},$$

where T_8_ and T_0_ represent the OD at time 0 h and after 8 h incubation. C_inh_ (inhibitory) of less than 0.4 is acceptable for probiotic candidate^38^.

### Assessment of cell surface hydrophobicity

Isolates were cultured in nutrient broth for 18 h and harvested pellets by centrifugation 3 min at 9400*g*, were washed and resuspended in 2 ml of PBS. To determine percentage of hydrophobicity, its optical density (OD) was measured and recorded as A_0_. After adding equal volume of Chloroform (Merck, Germany), Ethyl acetate (Merck, Germany) and Toluene (Merck, Germany) to bacterial suspension, blended them by vortexing for 5 min, and OD_600_ of aqueous phase was recorded as A_1_ after 30 min incubation at room temperature. The isolate adhering to solvents was estimated as below:

$${\text{Hydrophobicity }}\left( \% \right) = \left( {1 - \frac{A1}{{A0}}} \right) \times 100,$$

where A_0_ and A1 initial and final OD at 600 nm^45^.

### Auto-aggregation

For auto-aggregation test, after centrifugation of bacterial cells from overnight culture (nutrient broth, 37 °C), the pellet was washed and resuspended in buffer till absorbance of suspension reach to 0.3 ± 0.05 at 600 nm. Bacterial suspensions were vortexed for 10 s and OD_600_ of samples was recorded after incubation for 4 h and 24 h in 37 °C. Auto-aggregation was presented using the equation below:

$${\text{Auto - aggregation }}\left( \% \right) \, = \left( {1 - \frac{At}{{A0}}} \right) \times 100,$$

where A_t_ represented absorbance of samples in different times (4 or 24 h), A_0_ represented the absorbance at the beginning of the assay^54^.

### Bacterial adhesion assay

Adherence potential of candidate probiotic isolates was carried out with colonic adenocarcinoma cells that were obtained from Iranian Biological Resource Center (HT-29 IBRC C10097). First, 1 × 10^5^ cells/ml were seeded and incubated to obtained 80–90% confluency. Then cells in a 24-well plate were washed and medium was changed to antibiotic-free Dulbeccoʼs Modified Eagle Medium (DMEM) (Bioidea, Iran). Subsequently, 10^8^ cells/ml of test isolates were inoculated to cells in each well and incubated for 3 h (37 °C, 5% CO_2_). After incubated, three times washing of cells with PBS (Bioidea, Iran) was used for removing the non-adherent bacteria. Trypsinization of HT-29 monolayers by trypsin–EDTA solution (Bioidea, Iran) was done and finally, the cell attachment capacity was determined by Counting of adherent cells at time 0 (N0) and after 3 h (Nt) in triplicate on nutrient agar^55^.

$${\text{HT - 29 attachment }}\left( \% \right) \, = \left( {\log Nt/\log N0} \right) \times 100.$$

### Antibiotic resistance

Assessment of antibiotic resistance of *B.* *subtilis* isolates based on the recommendation of CLSI (Clinical and laboratory standards institute) was done by disc diffusion test. Briefly, approximately 10^8^ cfu/ml of overnight cultures were swabbed on the Mueller–Hinton Agar plates and antibiotic discs (Padtan Teb Co, Iran) containing tetracycline (30 μg), chloramphenicol (30 μg), erythromycin (15 μg), streptomycin (10 μg), vancomycin (30 μg), gentamycin (10 μg), clindamycin (2 μg) and penicillin (10 μg) were placed on the agar plates, and after 24 h incubation at 37 °C, sensitivity of bacteria was determined by measuring the diameter of inhibition zone^56^.

### DPPH scavenging assay

DPPH scavenging effect of cell free supernatants of two isolates was evaluated by mixing a volume of 100 μl filtrate culture with equal volume of DPPH solution (Merck, Germany) (0.2 mM) and left at 30 °C in darkness for 30 min. Deionized water was used as a control. Determination of absorbance at 517 nm using Synergy HTX multimode reader was done for measuring DPPH scavenging potency as below:

$${\text{DPPH}}\,{\text{scavenging}}\,{\text{activity }}\left( \% \right) \, = \left( {\frac{Ac - As}{{Ac}}} \right) \times 100,$$

A_c_: absorbance of control A_s_ : absorbance of samples^37,57^.

### Enterotoxin genes detection

DNA from *B.* *subtilis* isolates and *B.* *cereus* ATCC14579 as a positive control were extracted via the boiling method. In the next stage, PCR analyses were carried out to detect 6 enterotoxigenic genes. Table 6 show primer sequencers and PCR conditions^58^.

**Table 6:** Primer names and sequences, target size and PCR conditions for enterotoxin genes detection^58^.

Primers	Target size (bp)	Sequences (5′–3′)	Reaction conditions
hblA F hblA R	1154	AAGCAATGGAATACAATGGG AGAATCTAAATCATGCCACTGC	94 °C,2 min/(94 °C,60 s*56 °C,60 s*72 °C,120 s)35cycles /72 °C,5 min
hblC F hblC R	740	GATACCAATGTGGCAACTGC TTGAGACTGCTCGCTAGTTG	94 °C,2 min/(94 °C,60 s*58 °C,60 s*72 °C,120 s)35cycles /72 °C,5 min
hblD F hblD R	829	ACCGGTAACACTATTCATGC GAGTCCATATGCTTAGATGC	94 °C,2 min/(94 °C,60 s*58 °C,60 s*72 °C,120 s)35cycles /72 °C,5 min
nheA F nheA R	499	TACGCTAAGGAGGGGCA GTTTTTATTGCTTCATCGGCT	94 °C,2 min /(94 °C,60 s*56 °C,60 s*72 °C,120 s)35cycles /72 °C,5 min
nheB F nheB R	769	CTATCAGCACTTATGGCAG ACTCCTAGCGGTGTTCC	94 °C,2 min/(94 °C,60 s*54 °C,60 s*72 °C,120 s)35cycles /72 °C,5 min
nheC F nheC R	581	CGGTAGTGATTGCTGGG CAGCATTCGTACTTGCCAA	94 °C,2 min/(94 °C,60 s*58 °C,60 s*72 °C,120 s)35cycles /72 °C,5 min

### Statistical analysis

Analyzing results of three repetitions of experiments was done by IBM SPSS Statistics Software (version 26.0, SPSS Inc., USA) and presented as mean ± SD. Also, for finding significant difference (p ˂ 0.05) across means ANOVA analysis (One-way analysis of variance) followed by Post Hoc method (Duncan) was used.

## Data Availability

All data generated or analyzed during this study are included in this published article.
